# Supplementary figures and images for: Regulation of sleep by cholinergic neurons located outside the central brain in Drosophila
Source: PLoS Biol. 2023 Mar 2;21(3):e3002012. doi: 10.1371/journal.pbio.3002012 (PMC10013921; doi:10.1371/journal.pbio.3002012)

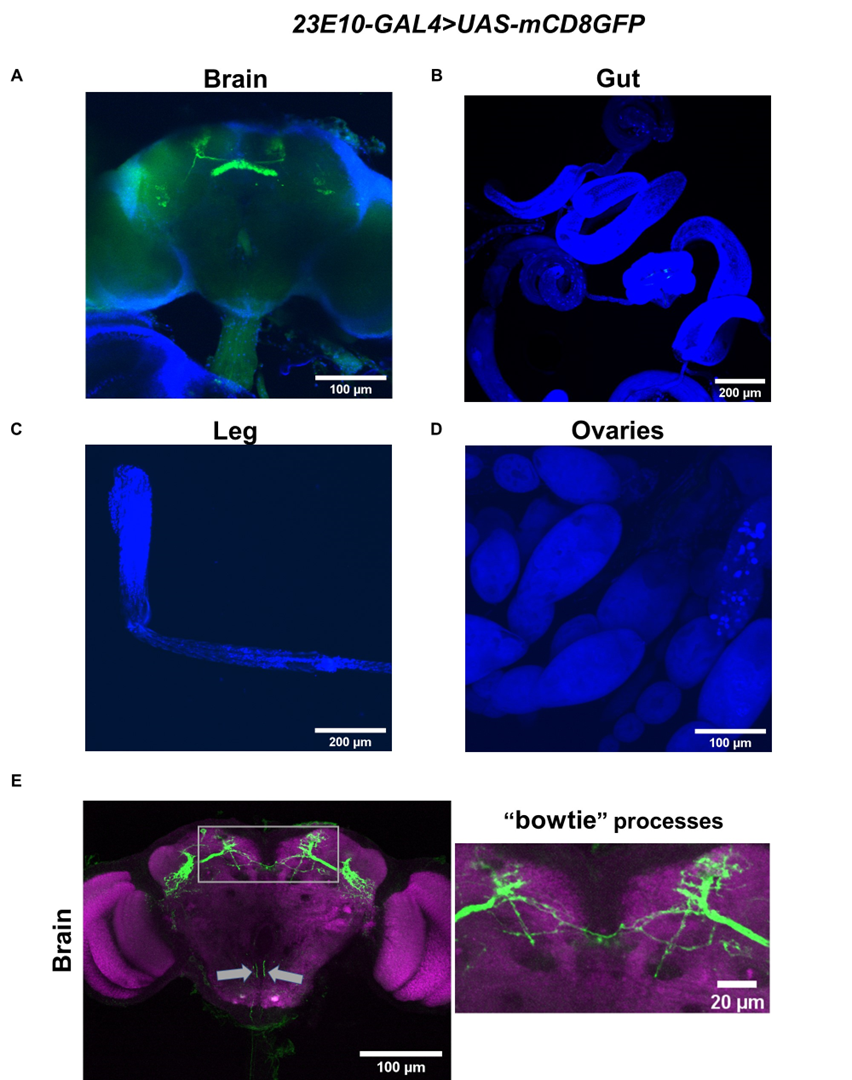

Supplement: S1 Fig — (A–D) Representative confocal stack images of adult tissues from 23E10-GAL4 > UAS-mCD8GFP female flies. GFP is expressed in the brain (A), but not the gut (B), leg (C), or ovaries (D). Tissue was dissected, fixed, and stained with DAPI. Green, GFP; blue, DAPI. (E) Representative confocal stacks of a female 23E10-GAL4>UAS-mCD8GFP brain centered on the “bowtie” processes from the VNC-SP neurons. Gray arrows indicate the processes of VNC-SP cells coming from the VNC. Area contained in the gray box is highlighted on the right. Green, anti-GFP; magenta, anti-nc82 (neuropile marker). (TIF) [file pbio.3002012.s001.tif]

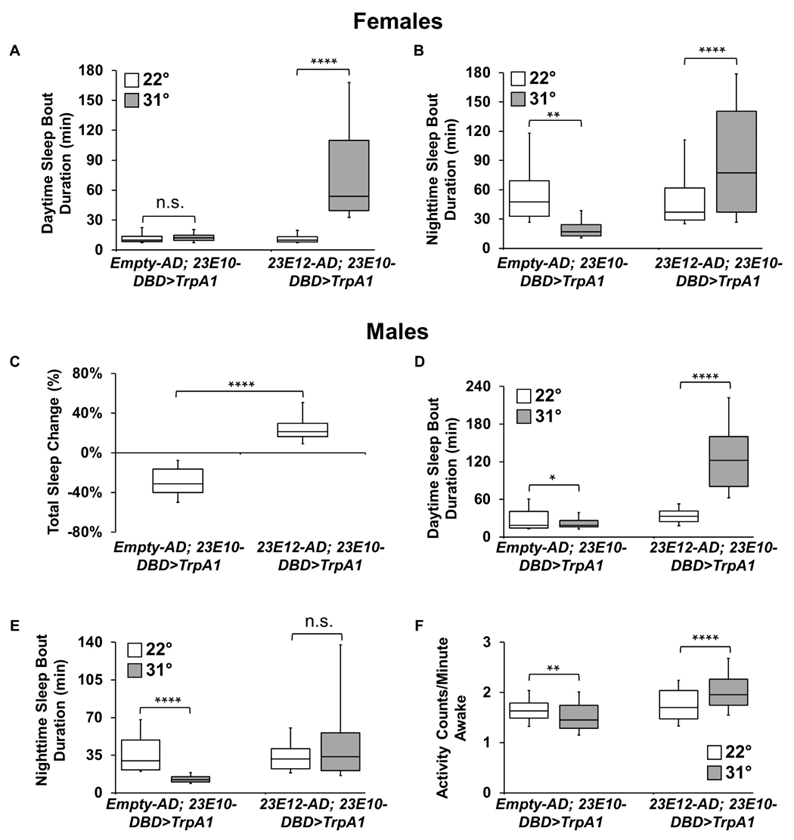

Supplement: S2 Fig — Additional data. (A) Box plots of daytime sleep bout duration (in minutes) for female flies presented in Fig 1M. Two-way repeated measures ANOVA followed by Sidak’s multiple comparisons test found that daytime sleep bout duration is significantly increased in 23E12-AD; 23E10-DBD>TrpA1 flies transferred to 31°C. ****P < 0.0001, n.s. = not significant, n = 44–51 flies per genotype. (B) Box plots of nighttime sleep bout duration (in minutes) for female flies presented in Fig 1M. Two-way repeated measures ANOVA followed by Sidak’s multiple comparisons test found that nighttime sleep bout duration is significantly increased in 23E12-AD; 23E10-DBD>TrpA1 flies transferred to 31°C. **P < 0.01, ****P < 0.0001, n = 44–51 flies per genotype. (C) Box plots of total sleep change in % ((total sleep on day 3-total sleep on day 2/total sleep on day 2) × 100) for male control (Empty-AD; 23E10-DBD) and 23E12-AD; 23E10-DBD flies expressing UAS-TrpA1. A two-tailed Mann–Whitney U test revealed that 23E12-AD; 23E10-DBD>TrpA1 flies increase sleep significantly more than control flies when transferred to 31°C. ****P < 0.0001, n = 43–46 flies per genotype. (D) Box plots of daytime sleep bout duration (in minutes) for flies presented in (C). Two-way repeated measures ANOVA followed by Sidak’s multiple comparisons test found that daytime sleep bout duration is significantly increased in 23E12-AD; 23E10-DBD>TrpA1 male flies transferred to 31°C. *P > 0.05, ****P < 0.0001, n = 43–46 flies per genotype. (E) Box plots of nighttime sleep bout duration (in minutes) for flies presented in (C). Two-way repeated measures ANOVA followed by Sidak’s multiple comparisons test found that nighttime sleep bout duration is not reduced in 23E12-AD; 23E10-DBD>TrpA1 flies transferred to 31°C, contrary to controls. ****P < 0.0001, n.s. = not significant, n = 43–46 flies per genotype. (F) Box plots of locomotor activity counts per minute awake for flies presented in (C). Two-way repeated measures ANOVA followed by [file pbio.3002012.s002.tif]

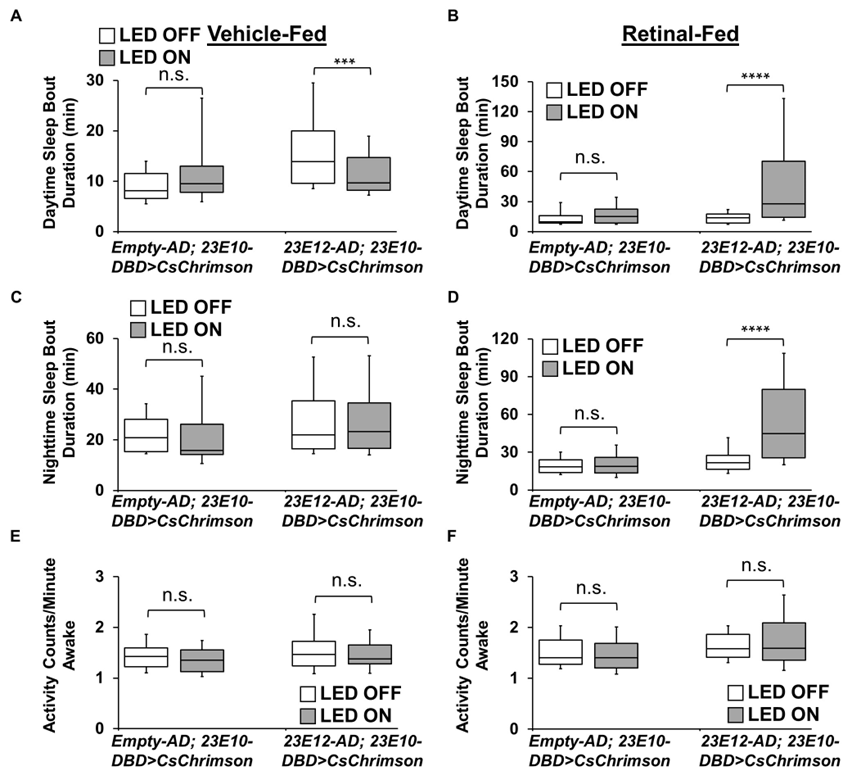

Supplement: S3 Fig — (A) Box plots of daytime sleep bout duration (in minutes) for vehicle-fed flies presented in Fig 1R. Two-way repeated measures ANOVA followed by Sidak’s multiple comparisons. ***P < 0.001, n.s. = not significant, n = 25–28 flies per genotype. (B) Box plots of daytime sleep bout duration (in minutes) for retinal-fed flies presented in Fig 1R. Two-way repeated measures ANOVA followed by Sidak’s multiple comparisons revealed that daytime sleep bout duration is significantly increased in activated 23E12-AD; 23E10-DBD>UAS-CsChrimson female flies. ****P < 0.0001, n.s. = not significant, n = 24–32 flies per genotype. (C) Box plots of nighttime sleep bout duration (in minutes) for vehicle-fed flies presented in Fig 1R. Two-way repeated measures ANOVA followed by Sidak’s multiple comparisons, n.s. = not significant, n = 25–28 flies per genotype. (D) Box plots of nighttime sleep bout duration (in minutes) for retinal-fed flies presented in Fig 1R. Two-way repeated measures ANOVA followed by Sidak’s multiple comparisons revealed that nighttime sleep bout duration is significantly increased in activated 23E12-AD; 23E10-DBD>UAS-CsChrimson female flies. ****P < 0.0001, n.s. = not significant, n = 24–32 flies per genotype. (E) Box plots of locomotor activity counts per minute awake for vehicle-fed flies presented in Fig 1R. Two-way repeated measures ANOVA followed by Sidak’s multiple comparisons, n.s. = not significant, n = 25–28 flies per genotype. (F) Box plots of locomotor activity counts per minute awake for retinal-fed flies presented in Fig 1R. Two-way repeated measures ANOVA followed by Sidak’s multiple comparisons, n.s. = not significant, n = 24–32 flies per genotype. The raw data underlying parts (A–F) can be found in S1 Data. (TIF) [file pbio.3002012.s003.tif]

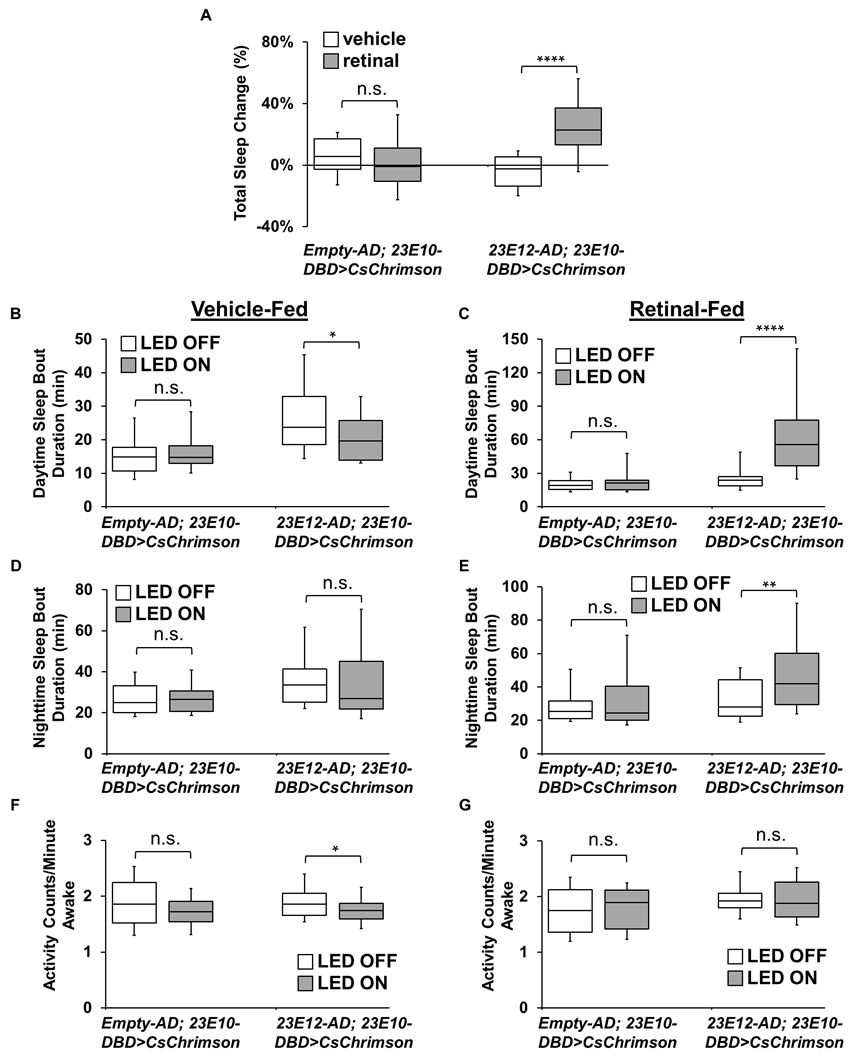

Supplement: S4 Fig — (A) Box plots of total sleep change in % ((total sleep on day 3-total sleep on day 2/total sleep on day 2) × 100) for control (Empty-AD; 23E10-DBD) and 23E12-AD; 23E10-DBD male flies expressing CsChrimson upon 627-nm LED stimulation. Two-way ANOVA followed by Sidak’s multiple comparisons revealed that total sleep is significantly increased in 23E12-AD; 23E10-DBD>UAS-CsChrimson male flies stimulated with 627-nm LEDs when compared with vehicle-fed flies. ****P < 0.0001, n.s. = not significant, n = 17–32 flies per genotype and condition. (B) Box plots of daytime sleep bout duration (in minutes) for vehicle-fed flies presented in (A). Two-way repeated measures ANOVA followed by Sidak’s multiple comparisons. *P < 0.05, n.s. = not significant, n = 17–32 flies per genotype. (C) Box plots of daytime sleep bout duration (in minutes) for retinal-fed flies presented in (A). Two-way repeated measures ANOVA followed by Sidak’s multiple comparisons revealed that daytime sleep bout duration is significantly increased in activated 23E12-AD; 23E10-DBD>UAS-CsChrimson male flies. ****P < 0.0001, n.s. = not significant, n = 20–30 flies per genotype. (D) Box plots of nighttime sleep bout duration (in minutes) for vehicle-fed flies presented in (A). Two-way repeated measures ANOVA followed by Sidak’s multiple comparisons, n.s. = not significant, n = 17–32 flies per genotype. (E) Box plots of nighttime sleep bout duration (in minutes) for retinal-fed flies presented in (A). Two-way repeated measures ANOVA followed by Sidak’s multiple comparisons revealed that nighttime sleep bout duration is significantly increased in activated 23E12-AD; 23E10-DBD>UAS-CsChrimson male flies. **P < 0.01, n.s. = not significant, n = 20–30 flies per genotype. (F) Box plots of locomotor activity counts per minute awake for vehicle-fed flies presented in (A). Two-way repeated measures ANOVA followed by Sidak’s multiple comparisons. *P < 0.05, n.s. = not significant, n = 17–32 flies per genotype. (G) Box plots o [file pbio.3002012.s004.tif]

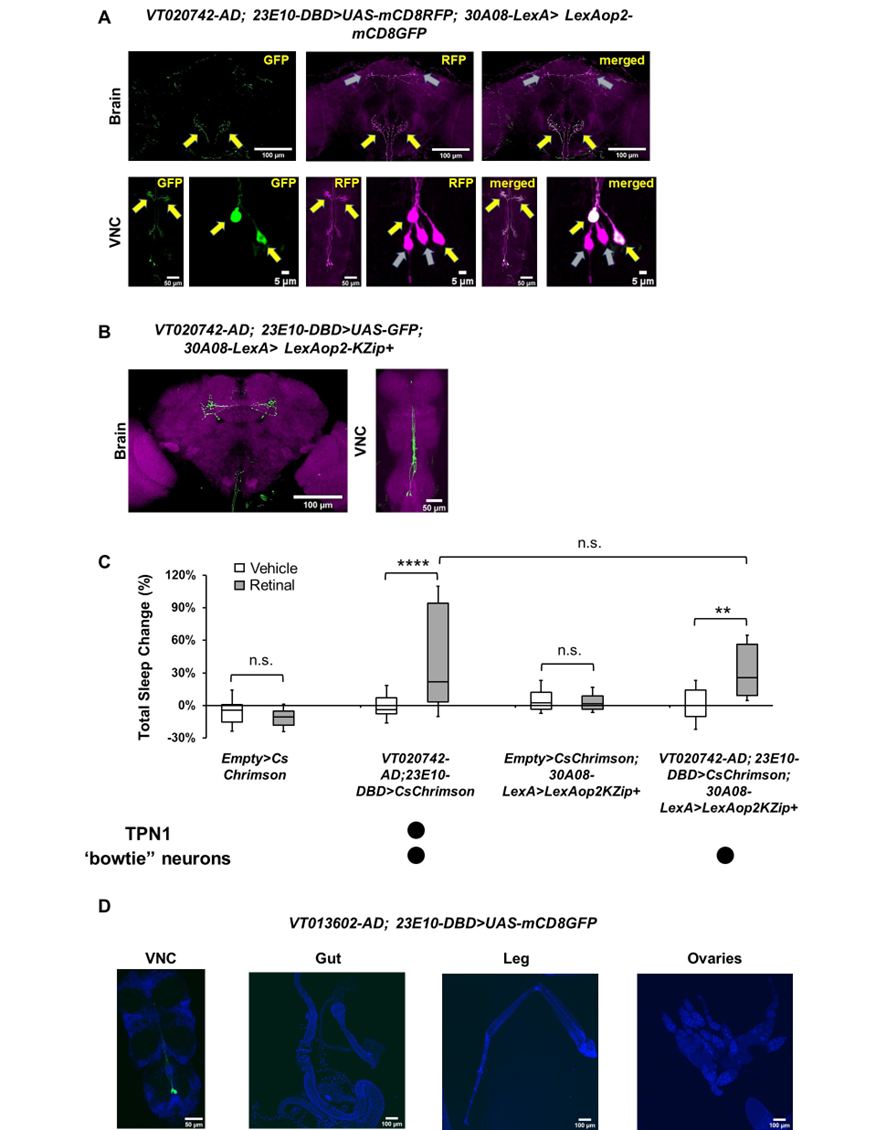

Supplement: S5 Fig — (A) Representative confocal stacks of a female 30A08-LexA>LexAop2-GFP; VT020742-AD; 23E10-DBD>UAS-RFP fly showing the brain, the VNC, and a magnified view of the cell bodies in the metathoracic ganglion. Yellow arrows show the TPN1 neurons and their processes in the VNC and in the brain and gray arrows, the VNC-SP neurons and their processes. TPN1 neurons are labeled by 30A08-LexA and VT020742-AD; 23E10-DBD while VNC-SP neurons are only present in VT020742-AD; 23E10-DBD. Green, anti-GFP; magenta, anti-RFP. (B) Representative confocal stacks of a female 30A08-LexA>LexAop2-KZip+; VT020742-AD; 23E10-DBD>UAS-GFP fly showing the brain and the VNC. The KZip+ repressor effectively remove expression in the TPN1 neurons leaving only the VNC-SP neurons. Green, anti-GFP; magenta, anti-nc82 (neuropile marker). (C) Box plots of total sleep change in % for Empty-AD; 23E10-DBD>UAS-CsChrimson, VT020742-AD; 23E10-DBD>UAS-CsChrimson, Empty-AD; 23E10-DBD>UAS-CsChrimson; 30A08-LexA> LexAop2-KZip+, and VT020742-AD; 23E10-DBD>UAS-CsChrimson; 30A08-LexA> LexAop2-KZip+ vehicle-fed and retinal-fed female flies upon 627-nm LED stimulation. Two-way ANOVA followed by Sidak’s multiple comparisons revealed that retinal-fed VT020742-AD; 23E10-DBD>UAS-CsChrimson and VT020742-AD; 23E10-DBD>UAS-CsChrimson; 30A08-LexA> LexAop2-KZip+ flies increase sleep significantly when stimulated with 627-nm LEDs when compared with vehicle-fed flies. Tukey’s multiple comparisons demonstrate that there is no difference in total sleep change between retinal-fed VT020742-AD; 23E10-DBD>UAS-CsChrimson and VT020742-AD; 23E10-DBD>UAS-CsChrimson; 30A08-LexA> LexAop2-KZip+ flies. **P < 0.01, ****P < 0.0001, n.s. = not significant, n = 17–31 flies per genotype and condition. (D) Representative confocal stack images of adult tissues from VT013602-AD; 23E10-DBD> UAS-mCD8GFP female flies. GFP is expressed in the VNC, but not the gut, leg, or ovaries. Tissue was dissected, fixed, and stained with DAPI. Green, GFP; blue, DAPI. T [file pbio.3002012.s005.tif]

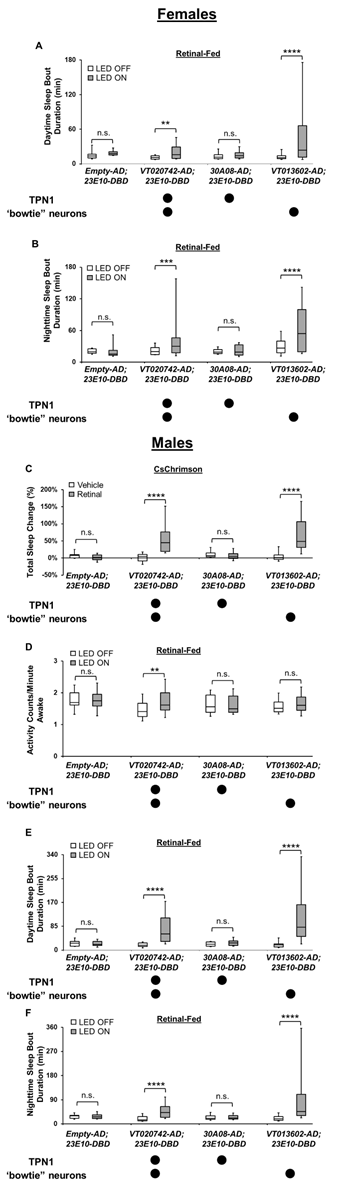

Supplement: S6 Fig — Additional data. (A) Box plots of daytime sleep bout duration (in minutes) for retinal-fed female flies presented in Fig 2K. Two-way repeated measures ANOVA followed by Sidak’s multiple comparisons indicates that daytime sleep bout duration is increased in retinal-fed VT020742-AD; 23E10-DBD>UAS-CsChrimson and VT013602-AD; 23E10-DBD>UAS-CsChrimson flies when stimulated with 627-nm LEDs. **P < 0.01, ****P < 0.0001, n.s. = not significant, n = 15–34 flies per genotype. (B) Box plots of nighttime sleep bout duration (in minutes) for retinal-fed female flies presented in Fig 2K. Two-way repeated measures ANOVA followed by Sidak’s multiple comparisons indicates that nighttime sleep bout duration is increased in retinal-fed VT020742-AD; 23E10-DBD>UAS-CsChrimson and VT013602-AD; 23E10-DBD>UAS-CsChrimson flies when stimulated with 627-nm LEDs. ***P < 0.001, ****P < 0.0001, n.s. = not significant, n = 15–34 flies per genotype. (C) Box plots of total sleep change in % for control (Empty-AD; 23E10-DBD>UAS-CsChrimson), VT020742-AD; 23E10-DBD>UAS-CsChrimson, 30A08-AD; 23E10-DBD>UAS-CsChrimson and VT013602-AD; 23E10-DBD>UAS-CsChrimson vehicle-fed and retinal-fed male flies upon 627-nm LED stimulation. Two-way ANOVA followed by Sidak’s multiple comparisons revealed that retinal-fed VT020742-AD; 23E10-DBD>UAS-CsChrimson and VT013602-AD; 23E10-DBD>UAS-CsChrimson flies increase sleep significantly when stimulated with 627-nm LEDs when compared with vehicle-fed flies. ****P < 0.0001, n.s. = not significant, n = 18–40 flies per genotype and condition. (D) Box plots of locomotor activity counts per minute awake for retinal-fed flies presented in (C). Two-way repeated measures ANOVA followed by Sidak’s multiple comparisons test show that locomotor activity per awake time is increased in VT020742-AD; 23E10-DBD>UAS-CsChrimson flies and is not affected in the other genotypes when the flies are stimulated with 627-nm LEDs. **P < 0.05, n.s. = not significant, n = 21–40 flies per genotype. (E) [file pbio.3002012.s006.tif]

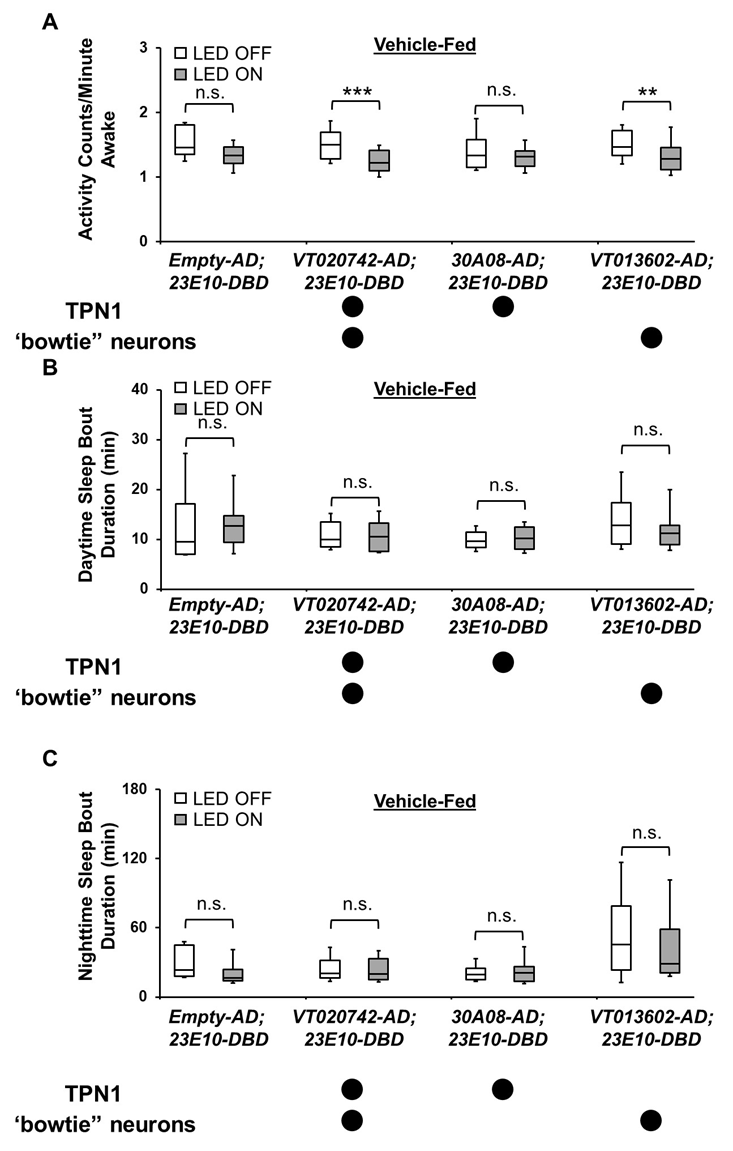

Supplement: S7 Fig — (A) Box plots of locomotor activity counts per minute awake for vehicle-fed flies presented in Fig 2K. Two-way repeated measures ANOVA followed by Sidak’s multiple comparisons test found that locomotor activity per awake time is reduced in vehicle-fed VT020742-AD; 23E10-DBD>UAS-CsChrimson and VT013602-AD; 23E10-DBD>UAS-CsChrimson flies that are stimulated with 627-nm LEDs. **P < 0.01, ***P < 0.001, n.s. = not significant, n = 13–32 flies per genotype. (B) Box plots of daytime sleep bout duration for vehicle-fed flies presented in Fig 2K. Two-way repeated measures ANOVA followed by Sidak’s multiple comparisons test found no difference in daytime sleep bout duration when the flies are stimulated with 627-nm LEDs, n.s. = not significant, n = 13–32 flies per genotype. (C) Box plots of nighttime sleep bout duration for vehicle-fed flies presented in Fig 2K. Two-way repeated measures ANOVA followed by Sidak’s multiple comparisons test show that nighttime sleep bout duration is not different when vehicle-fed flies are stimulated with 627-nm LEDs, n.s. = not significant, n = 13–32 flies per genotype. The raw data underlying parts (A–C) can be found in S1 Data. (TIF) [file pbio.3002012.s007.tif]

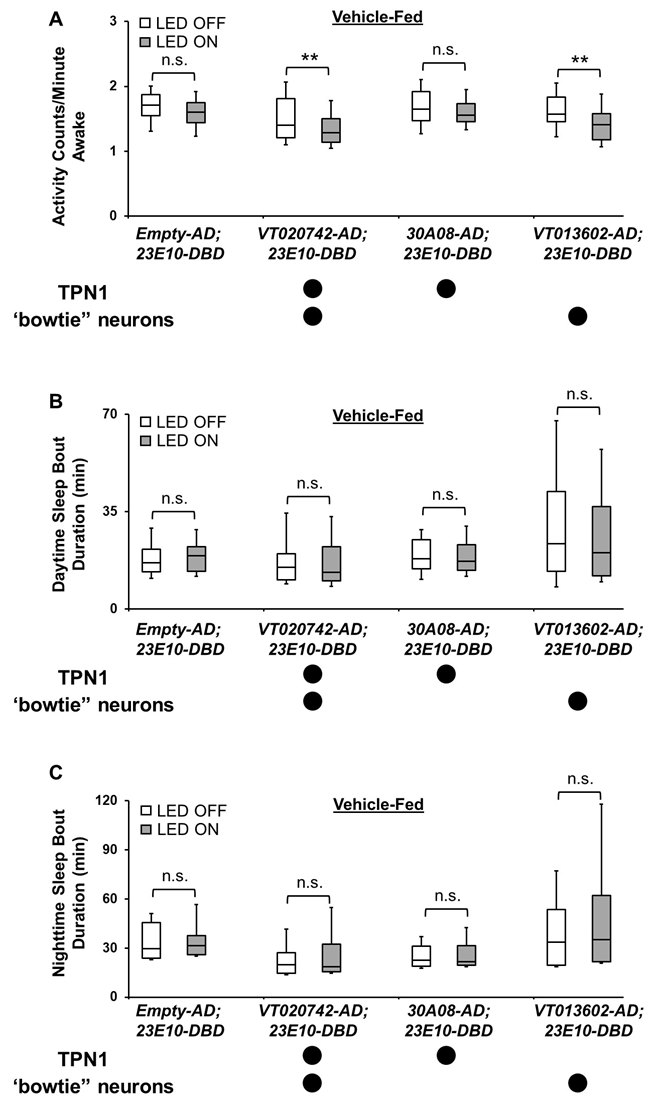

Supplement: S8 Fig — (A) Box plots of locomotor activity counts per minute awake for vehicle-fed flies presented in S6C Fig. Two-way repeated measures ANOVA followed by Sidak’s multiple comparisons test found that locomotor activity per awake time is reduced in vehicle-fed VT020742-AD; 23E10-DBD>UAS-CsChrimson and VT013602-AD; 23E10-DBD>UAS-CsChrimson flies that are stimulated with 627-nm LEDs. **P < 0.01, n.s. = not significant, n = 18–32 flies per genotype. (B) Box plots of daytime sleep bout duration for vehicle-fed flies presented in S6C Fig. Two-way repeated measures ANOVA followed by Sidak’s multiple comparisons test found no difference in daytime sleep bout duration when the flies are stimulated with 627-nm LEDs, n.s. = not significant, n = 18–32 flies per genotype. (C) Box plots of nighttime sleep bout duration for vehicle-fed flies presented in S6C Fig. Two-way repeated measures ANOVA followed by Sidak’s multiple comparisons test show that nighttime sleep bout duration is not increased when vehicle-fed flies are stimulated with 627-nm LEDs, n.s. = not significant, n = 18–32 flies per genotype. The raw data underlying parts (A–C) can be found in S1 Data. (TIF) [file pbio.3002012.s008.tif]

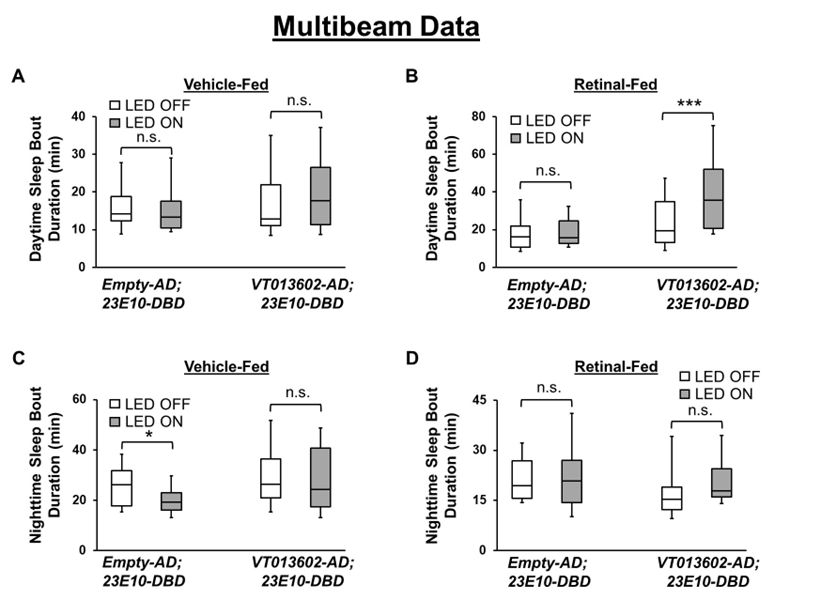

Supplement: S9 Fig — (A) Box plots of daytime sleep bout duration for multibeam analysis of vehicle-fed flies presented in Fig 2M. Two-way repeated measures ANOVA followed by Sidak’s multiple comparisons test found no difference in daytime sleep bout duration when the flies are stimulated with 627-nm LEDs, n.s. = not significant, n = 19–24 flies per genotype. (B) Box plots of daytime sleep bout duration for multibeam analysis of retinal-fed flies presented in Fig 2M. Two-way repeated measures ANOVA followed by Sidak’s multiple comparisons test show that daytime sleep bout duration is significantly increased in VT013602-AD; 23E10-DBD>UAS-CsChrimson flies stimulated with 627-nm LEDs. ***P < 0.001, n.s. = not significant, n = 23–24 flies per genotype. (C) Box plots of nighttime sleep bout duration for multibeam analysis of vehicle-fed flies presented in Fig 2M. Two-way repeated measures ANOVA followed by Sidak’s multiple comparisons test. *P < 0.05, n.s. = not significant, n = 19–24 flies per genotype. (D) Box plots of nighttime sleep bout duration for multibeam analysis of retinal-fed flies presented in Fig 2M. Two-way repeated measures ANOVA followed by Sidak’s multiple comparisons test, n.s. = not significant, n = 23–24 flies per genotype. The raw data underlying parts (A–D) can be found in S1 Data. (TIF) [file pbio.3002012.s009.tif]

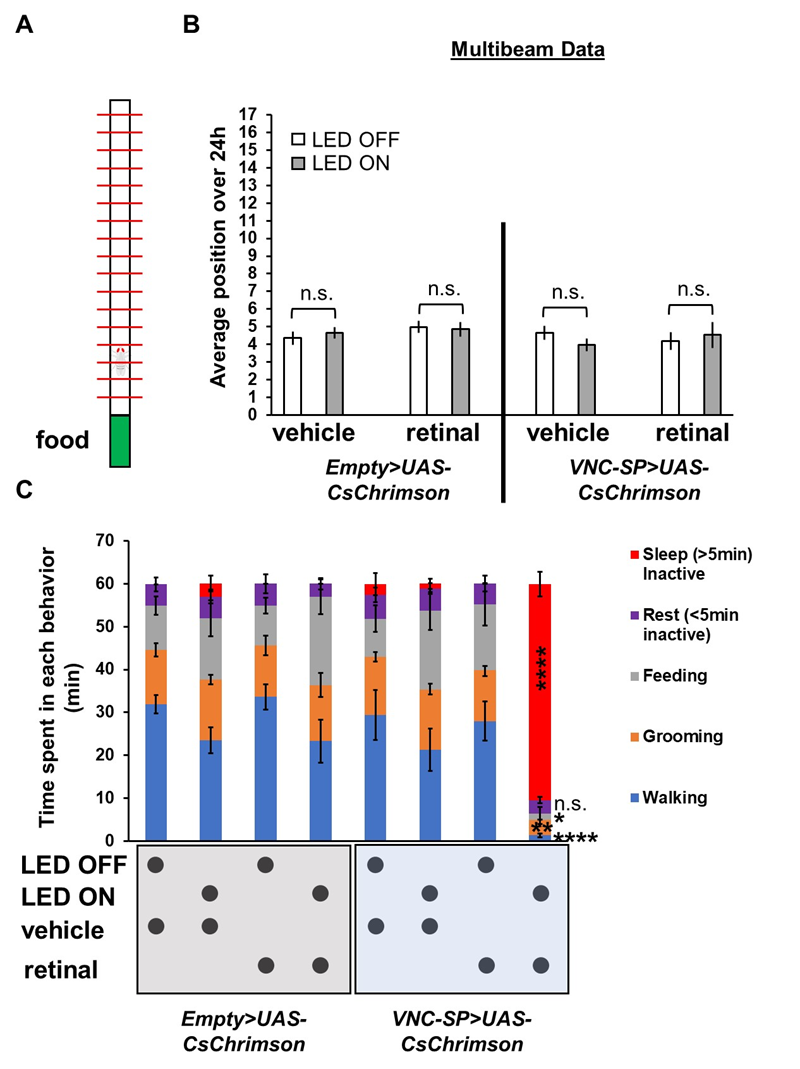

Supplement: S10 Fig — (A) Diagram of a multibeam tube with 17 individual infrared beams in red and fly for scale. (B) Graph of average fly beam position over 24 h. Each number on the y-axis represents a beam with the food positioned at 0. Beam position over 24 h was averaged at baseline with LED OFF (white bars) and activation day with LED ON (gray bars) for control Empty-AD; 23E10-DBD>UAS-CsChrimson and VNC-SP>UAS-CsChrimson flies fed vehicle or retinal. Three-way ANOVA followed by Tukey’s multiple comparisons test found no difference between the control and experimental fly position, n.s. = not significant, n = 19–24 flies per genotype and condition. (C) Video analysis of control and VNC-SP>UAS-CsChrimson female flies fed vehicle or retinal. Recording was performed at ZT1-2 for 1 h on consecutive days at baseline (LED OFF) and activation (LED ON). Behaviors were manually scored, and amount of time spent on each behavior over the hour is shown. Three-way ANOVA followed by Tukey’s multiple comparisons test found that VNC-SP>UAS-CsChrimson flies fed retinal with LED ON sleep significantly more than controls, ****P < 0.0001, but spend less time walking (****P < 0.0001), grooming (**P < 0.01), or feeding (*P < 0.05). Rest (periods of inactivity shorter than 5 min) are not different, n.s. = not significant, n = 5–7 flies for each genotype and condition. The raw data underlying parts (B) and (C) can be found in S1 Data. (TIF) [file pbio.3002012.s010.tif]

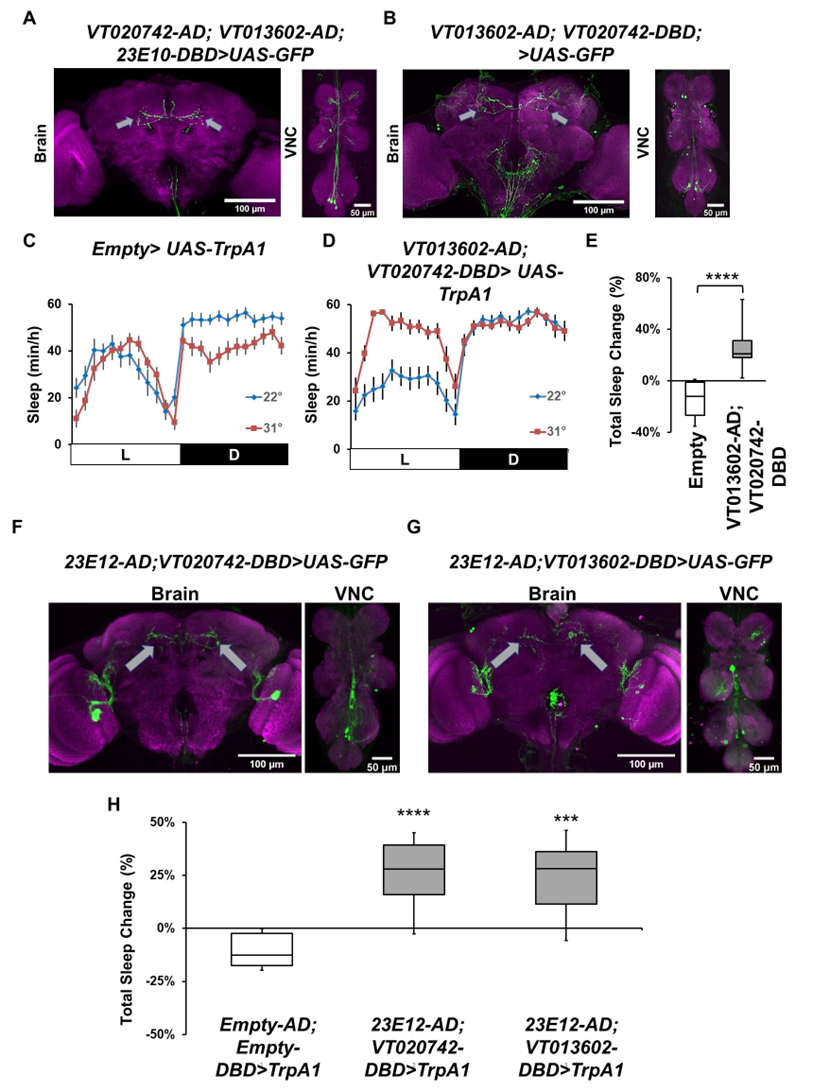

Supplement: S11 Fig — (A) Representative confocal stacks of a female VT013602-AD, VT020742-AD; 23E10-DBD>UAS-GFP fly showing the brain and the VNC. Gray arrows show the processes of VNC-SP neurons in the brain. Green, anti-GFP; magenta, anti-nc82 (neuropile marker). (B) Representative confocal stacks of a female VT013602-AD; VT020742-DBD>UAS-GFP fly showing the brain and the VNC. Gray arrows show the processes of VNC-SP neurons in the brain. Green, anti-GFP; magenta, anti-nc82 (neuropile marker). (C) Sleep profile in minutes of sleep per hour for Empty-AD; Empty-DBD>UAS-TrpA1 female flies maintained at 22°C (blue line) and transferred to 31°C (red line). (D) Sleep profile in minutes of sleep per hour for VT013602-AD; VT020742-DBD>UAS-TrpA1 female flies maintained at 22°C (blue line) and transferred to 31°C (red line). (E) Box plots of total sleep change in % for female control (Empty-AD; Empty-DBD) and VT013602-AD; VT020742-DBD flies expressing UAS-TrpA1. A two-tailed unpaired t test revealed that activating VT013602-AD; VT020742-DBD neurons significantly increases sleep compared with controls. ****P < 0.0001, n = 13–14 flies per genotype. (F) Representative confocal stacks of a female 23E12-AD; VT020742-DBD>UAS-GFP fly showing the brain and the VNC. Gray arrows show the processes of VNC-SP neurons in the brain. Green, anti-GFP; magenta, anti-nc82 (neuropile marker). (G) Representative confocal stacks of a female 23E12-AD; VT013602-DBD>UAS-GFP fly showing the brain and the VNC. Gray arrows show the processes of VNC-SP neurons in the brain. Green, anti-GFP; magenta, anti-nc82 (neuropile marker). (H) Box plots of total sleep change in % for female control (Empty-AD; Empty-DBD), 23E12-AD; VT020742-DBD, and 23E12-AD; VT013602-DBD flies expressing UAS-TrpA1. A one-way ANOVA followed by Tukey’s multiple comparisons demonstrate that activating neurons contained in the 23E12-AD; VT020742-DBD and 23E12-AD; VT013602-DBD line significantly increases sleep compared with controls. ***P < 0.001, ****P [file pbio.3002012.s011.tif]

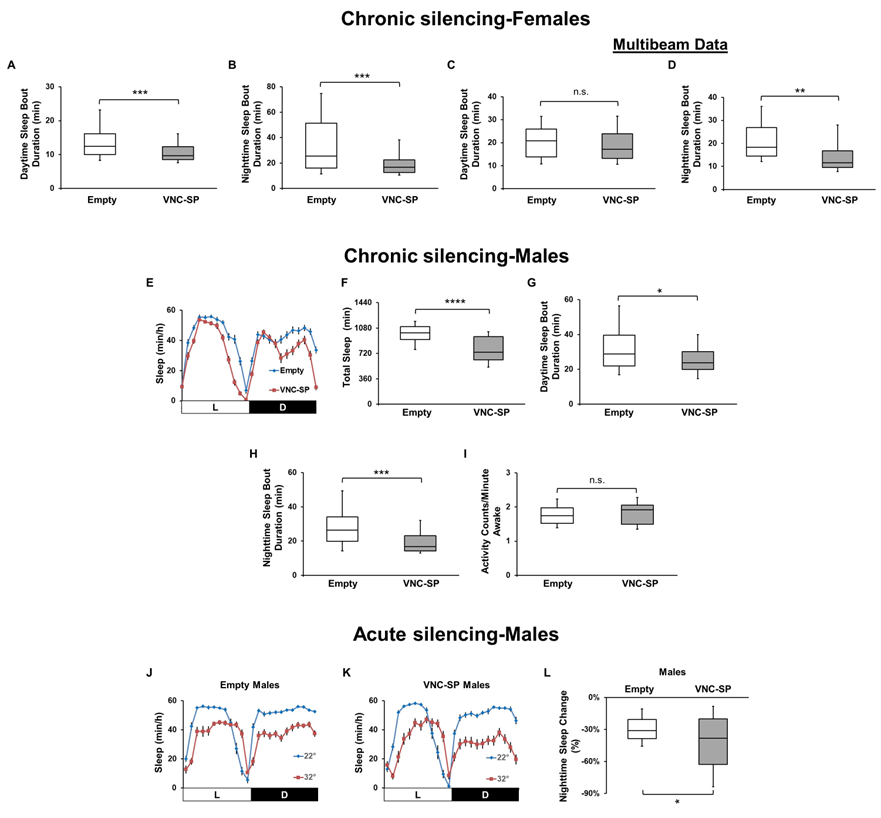

Supplement: S12 Fig — (A) Box plots of daytime sleep bout duration (in minutes) for flies presented in Fig 3A. Two-tailed Mann–Whitney U tests revealed that daytime sleep bout duration is significantly reduced in VNC-SP>Kir2.1 female flies compared to controls. ***P < 0.001, n = 58–60 flies per genotype. (B) Box plots of nighttime sleep bout duration (in minutes) for flies presented in Fig 3A. Two-tailed Mann–Whitney U tests revealed that nighttime sleep bout duration is significantly reduced in VNC-SP>Kir2.1 female compared to controls. ***P < 0.001, n = 58–60 flies per genotype. (C) Box plots of daytime sleep bout duration (in minutes) analyzed with the multibeam system for flies presented in Fig 3D. Two-tailed Mann–Whitney U tests revealed that daytime sleep bout duration is not different between VNC-SP>Kir2.1 female flies and controls, n.s. = not significant, n = 28–31 flies per genotype. (D) Box plots of nighttime sleep bout duration (in minutes) analyzed with the multibeam system for flies presented in Fig 3D. Two-tailed Mann–Whitney U tests revealed that nighttime sleep bout duration is significantly reduced in VNC-SP>Kir2.1 female compared to controls. **P < 0.01, n = 28–31 flies per genotype. (E) Sleep profile in minutes of sleep per hour for control (Empty-AD; 23E10-DBD>UAS-Kir2.1, blue line) and VNC-SP>Kir2.1 (VT013602-AD; 23E10-DBD>UAS-Kir2.1, red line) male flies. (F) Box plots of total sleep time (in minutes) for flies presented in (E). A two-tailed Mann–Whitney U test revealed that total sleep is significantly reduced in VNC-SP>Kir2.1 male flies compared to controls. ****P < 0.0001, n = 48 flies per genotype. (G) Box plots of daytime sleep bout duration (in minutes) for flies presented in (E). Two-tailed Mann–Whitney U tests revealed that daytime sleep bout duration is significantly reduced in VNC-SP>Kir2.1 and male flies compared to controls. *P < 0.05, n = 48 flies per genotype. (H) Box plots of nighttime sleep bout duration (in minutes) for flies presented in (E). Two-t [file pbio.3002012.s012.tif]

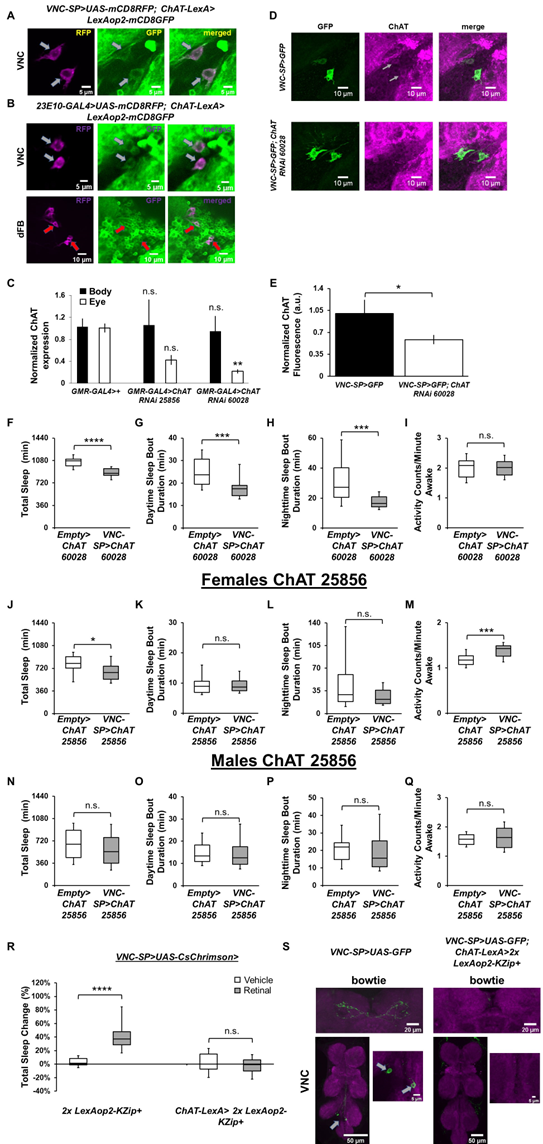

Supplement: S13 Fig — (A) Representative confocal stacks of a female ChAT-LexA>LexAop2-GFP; VT013602-AD; 23E10-DBD (VNC-SP)>UAS-RFP focusing on the cell bodies in the metathoracic ganglion of the VNC. Gray arrows show the VNC-SP neurons that are co-labeled by the ChAT-LexA driver. Green, anti-GFP; magenta, anti-RFP. (B) Representative confocal stacks of a female ChAT-LexA>LexAop2-GFP; 23E10-GAL4>UAS-RFP. Top panels, focusing on the cell bodies in the metathoracic ganglion of the VNC. Gray arrows show the VNC-SP neurons that are co-labeled by the ChAT-LexA driver. Bottom panels, focusing on dFB neurons. Red arrows show 23E10-GAL4 dFB neurons that are also labeled by ChAT-LexA. Green, anti-GFP; magenta, anti-RFP. (C) Quantification of ChAT knockdown efficiency by RNAi. qPCR was performed on the body and eyes of control flies (GMR-GAL4>+) and flies expressing ChAT RNAi (line 60028 and line 25856) in the eyes driven by GMR-GAL4 (GMR-GAL4>ChAT RNAi). Expression levels were normalized to control levels. Two-way ANOVA followed by Sidak’s multiple comparisons revealed that in GMR-GAL4>ChAT RNAi 60028 flies, ChAT levels are significantly reduced in the eyes but not in the body compared with controls. **P < 0.01, n.s. = not significant, n = 3–4 replicates per genotype. (D) ChAT immunostaining in VNC-SP>UAS-GFP (top panels) and VNC-SP>UAS-GFP; UAS-ChAT RNAi flies (bottom panels). Gray arrows point to neurons positive for GFP and ChAT. Green, anti-GFP; magenta, anti-ChAT. (E) Quantification of data presented in (D). A one-tailed unpaired t test revealed that expressing ChAT RNAi in VNC-SP neurons significantly reduce ChAT levels as measured with ChAT antibody staining. *P < 0.05, n = 14–17 VNC analyzed per genotype. (F) Box plots of total sleep time (in minutes) for control (Empty-AD; 23E10-DBD>ChAT 60028) and VNC-SP>ChAT 60028 male flies. A two-tailed Mann–Whitney U test revealed that VNC-SP> ChATRNAi male flies sleep significantly less than controls. ****P < 0.0001, n = 24–30 flies per genotype. ( [file pbio.3002012.s013.tif]

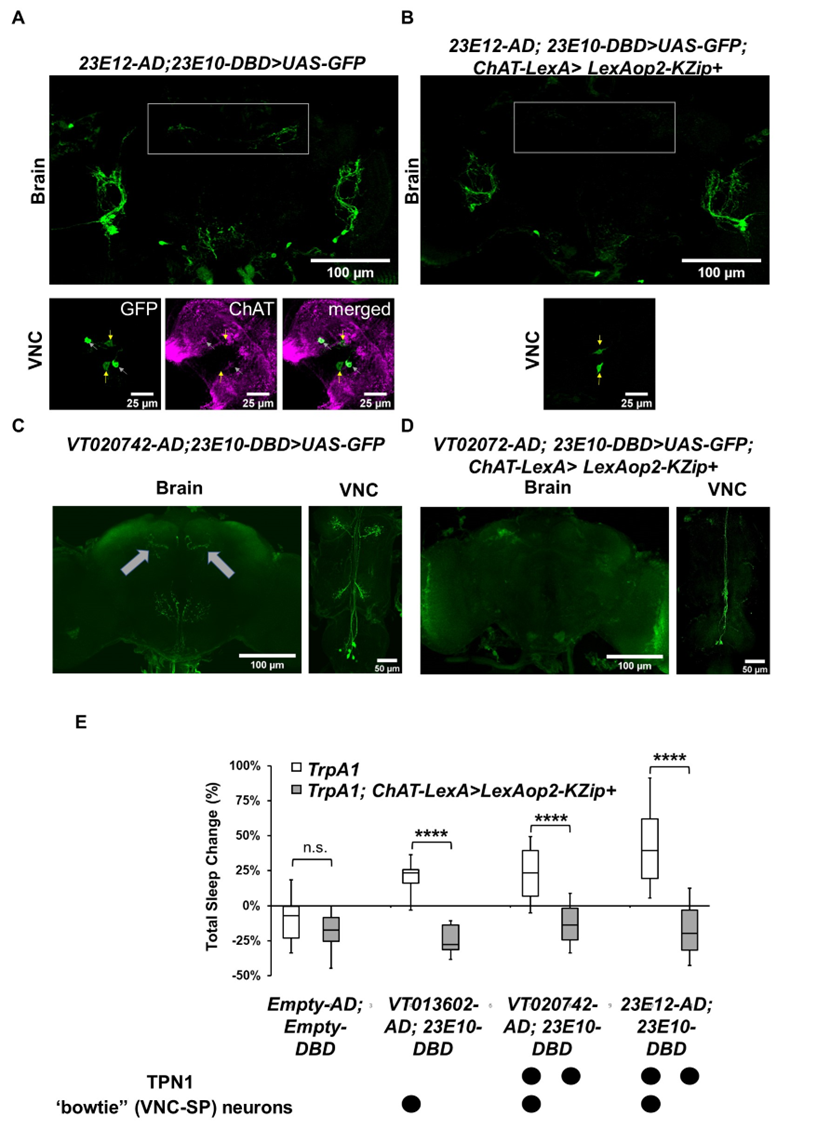

Supplement: S14 Fig — (A) Representative confocal stacks of a female 23E12-AD; 23E10-DBD>UAS-GFP fly showing the brain and the VNC. The gray rectangle shows the processes of VNC-SP neurons in the brain. Gray arrows show the cell bodies of VNC-SP neurons. Yellow arrows show the TPN1 neurons. Green, anti-GFP; magenta, anti-ChAT. (B) Representative confocal stacks of a female 23E12-AD; 23E10-DBD>UAS-GFP; ChAT-LexA> LexAop2KZip+ fly showing the brain and the VNC. The gray rectangle highlights the absence of processes of VNC-SP neurons in the brain. Yellow arrows show the TPN1 neurons. Green, anti-GFP. (C) Representative confocal stacks of a female VT020742-AD; 23E10-DBD>UAS-GFP fly showing the brain and the VNC. The gray arrows show the processes of VNC-SP neurons in the brain. Green, anti-GFP. (D) Representative confocal stacks of a female VT020742-AD; 23E10-DBD>UAS-GFP; ChAT-LexA> LexAop2KZip+ fly showing the brain and the VNC. Green, anti-GFP. (E) Box plots of total sleep change in % for female control (Empty-AD; Empty-DBD), VT013602-AD; 23E10-DBD, VT020742-AD; 23E10-DBD, and 23E12-AD; 23E10-DBD flies expressing UAS-TrpA1 or UAS-TrpA1; ChAT-LexA> LexAop2KZip+. A one-way ANOVA followed by Tukey’s multiple comparisons demonstrate that activating neurons contained in the 3 Split-GAL4 lines significantly increases sleep compared with controls and that removing expression in the VNC-SP neurons by expressing the KZip+ repressor in ChAT expressing neurons blocks this sleep increase. ****P < 0.0001, n.s. = not significant, n = 14–42 flies per genotype. The raw data underlying part (E) can be found in S1 Data. (TIF) [file pbio.3002012.s014.tif]

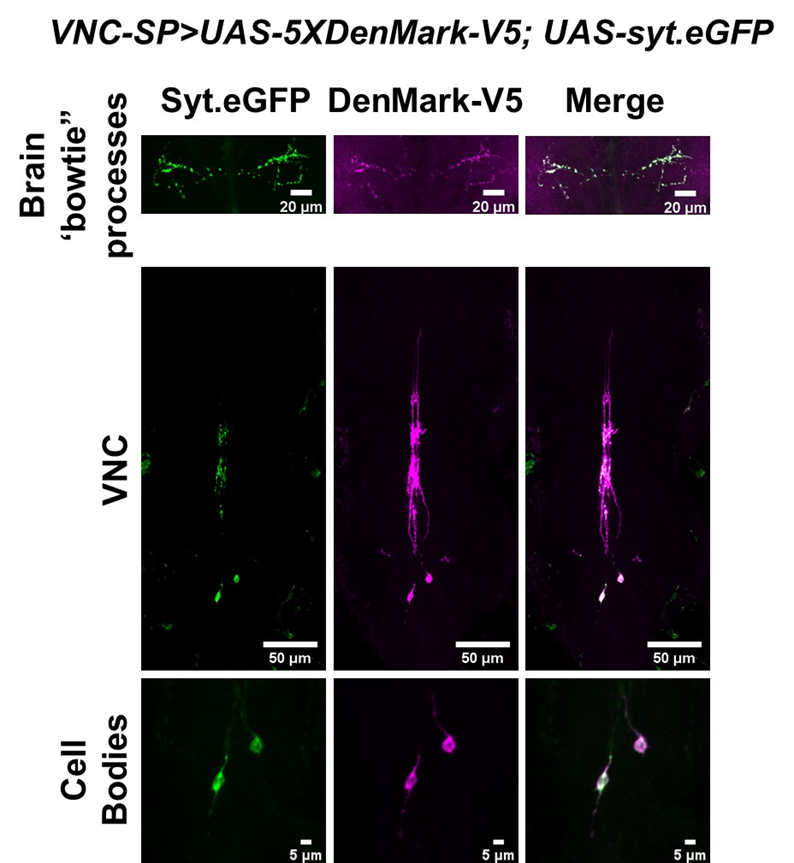

Supplement: S15 Fig — Representative confocal stacks of a female VT013602-AD; 23E10-DBD> UAS-5xDenMark-V5; UAS-syt.eGFP. Top panels, focusing on the brain “bowtie” processes. Middle panels, the VNC and bottom panels, a magnified view of the cell bodies area. Green, anti-GFP; magenta, anti-V5. (TIF) [file pbio.3002012.s015.tif]

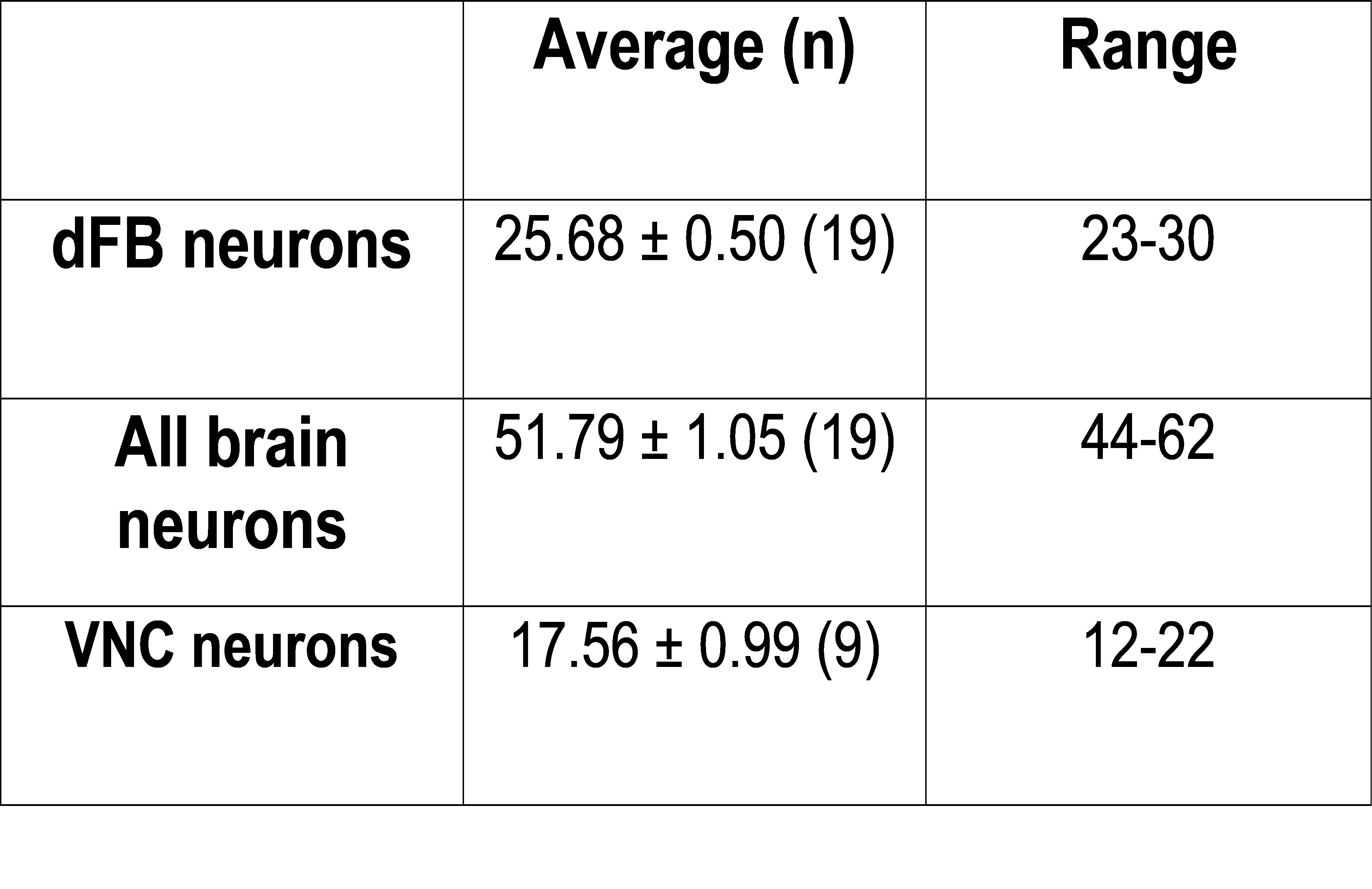

Supplement: S1 Table — Average number ± SEM and range of dFB neurons, all brain neurons and VNC neurons labeled in 23E10-GAL4>UAS-GFP female flies. The raw data underlying this table can be found in S1 Data. (TIF) [file pbio.3002012.s016.tif]
