# Supplementary material for: Regulation of sleep by cholinergic neurons located outside the central brain in Drosophila
Source: PLoS Biol. 2023 Mar 2;21(3):e3002012. doi: 10.1371/journal.pbio.3002012 (PMC10013921; doi:10.1371/journal.pbio.3002012)
Supplement: S2 Table — (DOCX) [file pbio.3002012.s017.docx]

| Genotype | Source or reference | Identifiers | Additional information |
| --- | --- | --- | --- |
| P{GMR23E10-GAL4}attP2 | Bloomington Drosophila Stock Center | RRID:BDSC_49032 |  |
| w[*]; P{y[+t7.7] w[+mC]=UAS-TrpA1(B).K}attP16 | Bloomington Drosophila Stock Center | RRID:BDSC_26263 |  |
| Canton-S | Laboratory of Paul Shaw |  |  |
| w[*]; P{y[+t7.7] w[+mC]=10XUAS-IVS-mCD8::GFP}attP40 | Bloomington Drosophila Stock Center | RRID:BDSC_32186 |  |
| w[*]; P{y[+t7.7] w[+mC]=10XUAS-IVS-mCD8::GFP}attP2 | Bloomington Drosophila Stock Center | RRID:BDSC_32185 |  |
| w[1118]; P{y[+t7.7] w[+mC]=23E10-GAL4.DBD}attP2/TM3 | Bloomington Drosophila Stock Center | RRID:BDSC_69269 |  |
| w[1118]; P{y[+t7.7] w[+mC]=p65.AD.Uw}attP40 | Bloomington Drosophila Stock Center | RRID:BDSC_71210 | Empty-AD |
| P{R23E12-p65.AD}attP40 | Bloomington Drosophila Stock Center | RRID:BDSC_70602 |  |
| w[1118]; P{y[+t7.7] w[+mC]=20XUAS-IVS-CsChrimson.mVenus}attP40 | Bloomington Drosophila Stock Center | RRID:BDSC_55135 |  |
| P{VT020742-p65.AD}attP40 | Bloomington Drosophila Stock Center | RRID:BDSC_73460 |  |
| P{R30A08-p65.AD}attP40 | Bloomington Drosophila Stock Center | RRID:BDSC_71007 |  |
| P{VT013602-p65.AD}attP40 | Bloomington Drosophila Stock Center | RRID:BDSC_74209 |  |
| P{GMR30A08-lexA}attP40 | Bloomington Drosophila Stock Center | RRID:BDSC_54756 |  |
| y[1] w[*]; P{y[+t7.7] w[+mC]=10XUAS-IVS-mCD8::RFP}attP18 P{y[+t7.7] w[+mC]=13XLexAop2-mCD8::GFP}attP8 | Bloomington Drosophila Stock Center | RRID:BDSC_32229 |  |
| w[*]; P{y[+t7.7] w[+mC]=13XLexAop2-KZip+.3XHA}su(Hw)attP5/CyO; TM6B, Tb[1]/MKRS | Bloomington Drosophila Stock Center | RRID:BDSC_76253 |  |
| w[*]; P{y[+t7.7] w[+mC]=13XLexAop2-KZip+.3XHA}attP2 | Bloomington Drosophila Stock Center | RRID:BDSC_76254 |  |
| w[*]; P{w[+mC]=UAS-Hsap\KCNJ2.EGFP}7 | Bloomington Drosophila Stock Center | RRID:BDSC_6595 | UAS-Kir2.1 |
| pJFRC100-20XUAS-TTS-Shibire-ts1-p10 in VK00005 | Laboratory of Gerry Rubin |  | UAS-Shi^ts1^ |
| GMR-GAL4 | Laboratory of Jeff Price |  |  |
| [P{GawB}elav[C155]](https://flybase.org/reports/FBti0002575.html) | Bloomington Drosophila Stock Center | RRID:BDSC_458 | elav-GAL4 |
| P{TRiP.HMC05021}attP40 | Bloomington Drosophila Stock Center | RRID:BDSC_60028 | Expresses dsRNA for RNAi of ChAT under UAS control |
| y[1] v[1]; P{y[+t7.7] v[+t1.8]=UAS-GFP.VALIUM10}attP2 | Bloomington Drosophila Stock Center | RRID:BDSC_35786 | Control RNAi line |
| w[1118]; P{w[+mC]=UAS-Dcr-2.D}2 | Bloomington Drosophila Stock Center | RRID:BDSC_24650 | UAS-Dicer2 on chromosome 2 |
| w[1118]; P{w[+mC]=UAS-Dcr-2.D}10 | Bloomington Drosophila Stock Center | RRID:BDSC_24651 | UAS-Dicer2 on chromosome 3 |
| w[1118]; P{y[+t7.7] w[+mC]=20XUAS-IVS-CsChrimson.mVenus}attP2 | Bloomington Drosophila Stock Center | RRID:BDSC_55136 |  |
| w[1118]; P{y[+t7.7] w[+mC]=20XUAS-IVS-CsChrimson.mVenus}attP18 | Bloomington Drosophila Stock Center | RRID:BDSC_55134 |  |
| w[*]; Mi{Trojan-lexA:QFAD.0}ChAT[MI04508-TlexA:QFAD.0] CG7715[MI04508-TlexA:QFAD.0-X]/TM6B, Tb[1] | Bloomington Drosophila Stock Center | RRID:BDSC_60319 | ChAT-LexA |
| w[1118]; P{w[+mC]=UAS-syt.eGFP}3 | Bloomington Drosophila Stock Center | RRID:BDSC_62138 |  |
| w[*]; P{y[+t7.7] w[+mC]=5XUAS-DenMark::smGdP-V5}su(Hw)attP5 | Bloomington Drosophila Stock Center | RRID:BDSC_6926 |  |
| P{TRiP.JF01877}attP2 (RNAi against ChAT) | Bloomington Drosophila Stock Center | RRID:BDSC_25856 | Expresses dsRNA for RNAi of ChAT under UAS control |
