# Supplementary material for: Regulation of sleep by cholinergic neurons located outside the central brain in Drosophila
Source: PLoS Biol. 2023 Mar 2;21(3):e3002012. doi: 10.1371/journal.pbio.3002012 (PMC10013921; doi:10.1371/journal.pbio.3002012)
Supplement: Attachment — Submitted filename: Response to Reviewers.docx [file pbio.3002012.s025.docx]

**Reviewer #1**: This study entails further mapping of the sleep system in Drosophila. The authors build upon a previous sleep system transgenic tool, 23E10-GAL4, which is expressed in fan-body neurons in the brain. By developing intersectional transgenic tools, Split-GAL4, they find that a small subset of neurons located outside the brain, in the ventral nerve cord, are also involved in sleep control. They go on to map their neurotransmitter expression and their basic neuroanatomy, and provide some evidence that the fan-body and the ventral nerve cord neurons control different aspects of sleep. Their analysis appears sound and the text is largely free from overinterpretations. Hence, there is no question that this study will be of great value for Drosophila sleep research. However, against the backdrop of the immense body of work on mammalian sleep, and given that sleep in mammals is also known to engage both brain and spinal cord circuits, it is unclear if the finding of ventral nerve cord sleep neurons in the fly is surprising and novel enough to appeal to the broad readership of PLoS Biology. A deeper understanding of the specific role of the ventral nerve cord sleep neurons in sleep control would have greatly strengthened the paper. In addition, there are some minor things that could improve this piece.

We thank the Reviewer for their comments. While there is a vast body of literature involving many brain structures in sleep regulation in mammalian and fly models, the role of spinal cord circuits is much less characterized. Most of the studies linking the spinal cord to sleep regulation have been performed in the context of spinal cord injury. Because our identified cells are both located in the fly equivalent of the spinal cord and are capable of modulating sleep in a healthy animal, we feel our discovery is sufficiently novel and provides a unique opportunity to study how brain-spinal cord circuitry regulates sleep in a genetically tractable model. Further work will investigate these interactions and the precise role of VNC-SP neurons in sleep regulation.

Introduction: The description of the fly sleep system is confusing, for instance rows 61-62 "Importantly, neurons that project to the dFB have emerged as a key sleep-regulating area." I would suggest adding a cartoon to figure 1 outlining what is known about the sleep system in flies.

There are multiple sleep-regulating circuits in the fly brain, these have been extensively described elsewhere. Importantly, our study was focused on the identification of individual sleep-regulating dFB neurons (as labeled by the 23E10-GAL4 driver) in the fly brain. We think that a cartoon that illustrates the multiple sleep-regulating regions in the fly brain would add confusion. However, we have created a cartoon in Fig. 1I that describes the goal of our screen. In addition, we have also changed the sentence referenced by the Reviewer to “In addition, dFB neurons have emerged as a key sleep-regulating area as their activation promotes sleep while their neuronal silencing reduces sleep (14-22).”

Abstract: The abstract is perhaps a bit too "23E10-GAL4-centered". The main finding of this study is that they identify neurons in the nerve cord that are involved in sleep, not that they are a subset of 23E10-GAL4-expressing neurons, which one would gather is only meaningful to a handful of labs around the world. The same applies to the Introduction, rows 81-84.

We have modified the abstract to make it less 23E10-GAL4 centric.

The section in rows 81-84 has also been flagged by Reviewer 2 and we have decided to remove it from the Introduction as it is, as the Reviewer suggested, only meaningful for a minority of labs and does not affect our main finding.

Introduction and/or Results, first section: It would be good if they define how they measure fly sleep. It's outlined in the Methods, but should be described here as well.

We have modified the results section line 92 to read “Individual flies were loaded in Drosophila Activity Monitors (DAM2, Trikinetics) and assessed for two baseline days at 22°C, before raising the temperature to 31°C for 24h on day 3 (Fig 1A) and sleep was defined as any period of inactivity lasting for at least 5 min, as previously described (30). “

Results, rows 165-166: They should provide some info as to how they arrived at the intersectional Split-GAL4 tool used herein.

For clarification, we changed the section describing our screen (line 165) to “In this screen, we created new Split-GAL4 drivers by combining AD and DBD lines that are putatively expressing in the dFB, as assessed by the expression pattern of their corresponding GAL4 lines. The complete description and results of this ongoing Split-GAL4 dFB-based screen are beyond the scope of this manuscript and will be described elsewhere. However, when performing this screen, we identified a line (23E12-AD;23E10-DBD) that strongly promotes sleep when thermogenetically activated with the TrpA1 channel (Fig 1K and 1L) when compared with an enhancer-less (empty) AD construct combined with 23E10-DBD (Fig 1J and 1L).”

Fig 5: Outline cells in B and C as well.

We have added arrows in Fig.5 B and C (now Fig. 4 B and C) to highlight VNC-SP neurons.

**Reviewer #2**: All in all this is very comprehensive and incisive study leading to the identification of a precise pair of neurons in the Drosophila CNS, but outside the brain, which has a clear role in sleep promotion but not in sleep rebound. The experimental work is rigorous and the logic generally sound. I think the advance is clear and two-fold: first it provides a stark example of the limitations of "looking where the light is". Meaning if you only look in the brain, you will focus on brain neurons only. The second advance is to identify a non-brain neuronal cell class that potently affects sleep state (not waking state) in both loss of function and gain of function conditions. As suggested by the authors, it implies significant flow of information from the periphery to the CNS to influence the movement into sleep. This work should have a substantial influence on the sleep field (but also other fields) in the fly model system. I have only minor comments that may help improve this excellent manuscript.

We thank the Reviewer for their very supportive and enthusiastic comments.

Line 79

The authors write:

"Strikingly…"

Suggest removing such descriptors and leaving impressions about the information for the reader to form.

We agree with the Reviewer and have removed Strikingly, the sentence now reads “We report here the identification of two non-dFB 23E10-GAL4 expressing, sleep-regulating cholinergic neurons located in the Ventral Nerve Cord (VNC), the fly equivalent to the spinal cord.”

Line 81

The authors write:

"Thus, our work reveals that an extensively used "dFB-specific" GAL4 driver contains non-dFB sleep-promoting neurons, raising important questions about the role of the dFB in sleep regulation and highlighting the importance of using specific tools in behavioral manipulations."

These are important considerations and I agree the Gal4 lines contains more elements than the dFB that appear to promote sleep. However, I'm not sure I understand why this results calls into question the role of the dFB in sleep regulation? Further, beyond using three different drivers that shared dFB expression, the original report (Donlea et al) made the same conclusion:

"In contrast, the expression pattern of these three drivers did not show prominent overlap in neurons in other brain regions (fig. S2) (12). Thus, although we cannot formally exclude a role for brain regions outside the dorsal FB, it is likely that the dorsal FB plays a role in regulating quiescence."

The present work therefore adds important new information that was not previously excluded; it does not disprove conclusions previously expressed. I suggest the language be modified to reflect that more accurately.

The Reviewer is right about the Donlea *et al*., 2011 study. In their work, the authors used three different GAL4 lines that share expression in the dFB and demonstrate that they can induce sleep when activating neurons contained within these 3 GAL4 drivers. What we were trying to highlight in this sentence is the essential need to use genetic tools that are as clean as possible. We note that the 3 drivers used in the 2011 paper cited above are not clean at all. However, the Reviewer is right, our work does not invalidate previous conclusions on the dFB.

We want to reiterate that the identification of VNC-SP neurons was a surprise to us. There is a vast amount of data supporting a role for dFB neurons in sleep regulation. The original goal of our screen was to identify the contribution of individual dFB neurons (as labelled with the 23E10-GAL4 driver, which is the most widely used “dFB-specific” tool).

Since that sentence was also flagged by Reviewer 1, we have decided to remove it from the Introduction and we have modified the sentence just before to “We report here the identification of two non-dFB 23E10-GAL4 expressing, sleep-regulating cholinergic neurons located in the Ventral Nerve Cord (VNC), the fly equivalent to the spinal cord.”

line 150

The authors write:

"To clarify the expression pattern of 23E10-GAL4, we expressed GFP under its control and identified more than 50 GFP-positive neurons in the brain, only half of which are dFB neurons (Fig 1G and 1H, and Table S1). In addition, 23E10-GAL4 expresses in about 18 neurons in the VNC …"

Was expression outside the CNS examined?

We thank the Reviewer for this suggestion and have examined 23E10-GAL4 expression outside the CNS. We found that 23E10-GAL4 does not express outside the CNS (Fig. S1B-S1E).

line 165

The authors write:

"The description and results of this Split-GAL4 screen are beyond the scope of this manuscript and will be described elsewhere."

I recommend providing a description of the screen with this report. Publishing has moved beyond the days of including "unpublished results".

We understand the Reviewer’s position on the importance of publishing the entirety of our screen. However, it is important to know that while the first objective of our work was to identify the role of individual 23E10-GAL4 dFB neurons in sleep regulation, the finding of non-dFB 23E10-GAL4 sleep-promoting cells was surprising and exciting enough to shift our focus almost exclusively on the characterization of VNC-SP neurons. Thus, we believe that the dFB part of our screen is not complete yet and that it would be premature to publish alongside the current investigation of VNC-SP neurons. Furthermore, we feel that adding these data would confound the message of the current study on VNC-SP neurons. For example, at this stage we cannot say for sure whether any 23E10-GAL4 dFB neurons regulate baseline sleep. Importantly, we are still in the process of creating tools that would undeniably assess the involvement of 23E10-GAL4 dFB neurons in sleep regulation. However, we believe that the dFB must be involved in some aspects of sleep regulation based on the extensive amount of published data supporting a role for dFB neurons. Thus, it is essential that we do the best possible job on our dFB screen so that we can reconcile our work with the published literature. With this in mind we feel that adding these data would be detrimental to the current study on VNC-SP neurons.

line 194

The authors write

"Two of these neurons show a specific pattern of expression, with brain processes located in the superior medial protocerebrum …:

Are the bowtie processes in the protocerebrum visible in 23E10-Gal4>GFP brains?

Yes, the bowtie processes are visible in the brain of 23E10-GAL4>GFP flies (Movie S1). However, they are located very close to the processes of dFB neurons, making them difficult to see in a confocal stack image of the entire brain. Thus, we have added a representative image focused only on these processes in a 23E10-GAL4>GFP brains (see below and Fig S1A).

line 325

The authors write:

"Optogenetic activation of 30A08-AD; 23E10-DBD neurons has no effect on sleep (Fig 3K-3N 326 and Fig S4A-S4D), indicating that the sleep-promoting capacity of VT020742-AD; 23E10-DBD 327 most likely lies within the two "bowtie" neurons."

Strictly speaking, it says TPN1 neurons are not sufficient. It says nothing about the sufficiency of the bowtie neurons. The experiments described later on line 333 do provide that demonstration, so I recommend modifying the text in lines 325-327.

We thank the Reviewer for this suggestion and have modified the sentence to now read “Optogenetic activation of 30A08-AD; 23E10-DBD neurons has no effect on sleep (Fig 2K, Fig S6A and S6B and Fig S6C-S6F for males), indicating that activation of TPN1 neurons is not sufficient to induce sleep.”

line 467

The authors write:

"…RNAi lines for ChAT. Out of these two lines, only one (60028) led to a significant reduction in 468 ChAT levels, as judged by qPCR and immunocytochemistry, and was used in our behavioral 469 studies (Fig S7C-S7E)."

It would be useful to assess behavior with the 2nd RNAi to ask if there is a correlation. I do not like to ask for additional experiments generally, but this one appears of limited scope. I could strengthen the conclusion linking VNC-SP action via cholinergic transmission.

We have included the quantification of RNAi efficiency for the second line (25856) in Fig S12C as well as behavioral data using this RNAi line in Fig S12J-S12Q. Note that line 25856 is trending towards a capacity to reduce ChAT levels, however it does not reach significance when compared with controls (Two-way ANOVA followed by Sidak’s multiple comparisons, p=0.0696). Not surprisingly, expressing this line in VNC-SP neurons has a much milder effect (if any) on sleep compared with line 60028. Namely, we only see a reduction in total sleep in female flies. No other sleep parameters are affected. In addition, we see no significant effects in males. These data are in marked contrast with the data obtained with line 60028, which has a strong impact on multiple sleep parameters in males and females.

**Reviewer #3**: This is an excellent piece of work and well presented. The field has reached dangerous level of sloppiness and papers like this one are a stark reminder that we all ought to be better. The genetics is fantastic and the data presentation is careful.

There are only two comments that I need to make.

We thank the Reviewer for his very enthusiastic comment about our genetic approach.

Major comment (decision left to the editor).

The authors show how important it is to be careful and considerate in drawing conclusions based on approximate genetics. They do an excellent job in demonstrating that 23E10 neurons include a small but important population of activity regulating neurons in the VNC. They, however, take a suboptimal approach themselves when it comes to behavioural characterization, relying exclusively on the DAM system. The DAM system is obsolete. It should no longer be used for ANY sleep paper, let alone for a beautiful paper like this one whose purpose is to correct other people's work. I would like to see the key experiments reproduced using a more appropriate system. My recommendation would be to employ video tracking or, as a second best , a more modern multi-beam system.

First, we again thank the Reviewer for his comment. We agree that due to the nature of the DAM system, it is in theory possible that periods of inactivity that are not sleep, and episodes of grooming and feeding behaviors could be falsely labeled as sleep using the DAM2 single-beam monitors. We want to emphasize though that the DAM system is still widely used by most sleep lab and that to our knowledge, sleep data obtained with the single-beam DAM2 system has never been invalidated by video-tracking methods or multibeam systems. In addition, we would like to point out that to perform a large-scale screen like ours, the DAM system is still extremely valuable as it provides a way to analyze sleep in thousands of flies on any given day, something that is more complex using other methods.

However, to fully characterize the role of VNC-SP neurons in sleep regulation, we have repeated key experiments using the Trikinetics multibeam MB5 system. We have added in this resubmission an analysis of sleep data obtained when optogenetically activating VNC-SP neurons using the multibeam system. These data can be found in Fig 2M (and below) and Fig S9 and confirm our DAM single-beam data, ie activation of VNC-SP neurons increases total sleep and sleep consolidation during the day.

Furthermore, silencing VNC-SP neurons reduces sleep and sleep consolidation using the multi-beam system (Fig 3D and Fig S11C and S11D), confirming our previously obtained DAM2 single-beam data.

Importantly, the multibeam system provides us with a means to analyze the fly’s individual positions in the tube. This analysis reveals that LED activated VNC-SP>Chrimson flies are not located closer to the food than their non-activated siblings or controls. Thus, it is unlikely that activation of VNC-SP neurons increases the duration of feeding (Fig S10B).

To further rule out the possibility that increasing the activity of VNC-SP neurons induces a behavior that could falsely be labeled as sleep (like grooming or feeding) using the DAM system, we decided to employ a video analysis approach to more closely look at the behavior. As seen in Fig S10C and below, activating VNC-SP neurons increases the duration of sleep and reduces the duration of walking, grooming, and feeding behaviors. Thus, we conclude that activating VNC-SP neurons induces sleep.

My lab recently published an experiment very similar to the one shown in Figure 1BCD of this paper using ethoscope-based videotracking (see French et al Nature 2021, Supplementary Figure 5 - the only difference is the activation temperature 29C vs 31C). In that experiment, we showed that 23E10 activation using trpA1 does not lead to an increase in sleep but a decrease in sleep caused by what is likely to be an increase in feeding. Keep in mind that any manipulation that increases feeding activity will be misunderstood as an increase in sleep by the DAM system because it will result in less beam crossing activity. I commend the fact that you at least use arousal threshold as a way to improve your findings (Fig 3O) but be aware that arousal threshold will be changed by feeding activity too (a fly won't necessarily react to mechanical stimuli while it's eating).

We are aware of the data presented in French et al Nature 2021, Supplementary Figure 5. However, we believe that these data do not in any way contradict or invalidate our results. From personal experience, we know that the temperature used to activate the TrpA1 channel can have a huge impact on the behavior analyzed. In addition, different studies have used temperatures ranging from 27C to 31C when using TrpA1 as an effector. The accepted view is that the higher the temperature, the stronger the activation. In our work, we are using a temperature of 31C to ensure activation.

Regarding the French et al Nature 2021 data, we agree that using their videotracking system the authors do not see an increase in sleep when maintaining 23E10-GAL4>TrpA1 flies at 29C. However, the authors do not provide any DAM system data using the same genotype treated in the same way. Thus, it is impossible to compare our results with their study. Several important questions need to be addressed to compare our work to their study. 1) What would sleep look like if the authors performed the same experiment in the DAM system? 2) Conversely, what would sleep look like in 23E10-GAL4>TrpA1 flies maintained at 31C using videotracking? 3) Is it possible that in their study, the authors did not see an increase in sleep at 29C when using videotracking because this temperature is simply not sufficient to reliably activate 23E10-GAL4 neurons? To try to answer some of these questions, we have conducted an experiment monitoring sleep in 23E10-GAL4>TrpA1 flies over three consecutive days using the DAM system (see below). Day 1 at 22C (baseline day, blue line), Day 2 at 29C (red line) and Day 3 at 31C (green line). The graph below demonstrates that in our laboratory, 23E10-GAL4>TrpA1 flies maintained at 29C do not increase sleep in the DAM single-beam system compared with baseline day. However, transferring the same flies to 31C lead to an increase in sleep. Taken together with our response to the previous comments, we are confident that we have demonstrated that the data we have obtained using the DAM system is real sleep and not another behavior (grooming or feeding) that is mislabeled as sleep.

Minor comments (decision left to the authors).

1. Personally, I do not place any value in any analysis of sleep bouts (duration, numbers) when data are collected with DAMs because they simply cannot offer enough resolution for this kind of secondary analysis. This paper is full of those so if things must stay as they are I would recommend moving them to supplementary. That would also allow to join Figure 1 and 2 into one figure. I think, in general, the paper can benefit from being shortened. It has a simple message that gets a bit lost in a long narrative.

We thank the Reviewer for this suggestion, and we have joined Figure 1 and 2 together and moved some of the analysis into supplementary Figures. However, we want to emphasize that the multibeam data we collected confirms that VNC-SP neurons modulate sleep consolidation.

2.The combination of split GAL4 line is difficult to follow and one will easily get lost. You can solve this in multiple ways: 1) having cartoons showing "which line expresses where" in Figure 3; 2) giving a human-readable nickname to the split GAL4 combinations, in the figure and the text. I recommend doing both.

We thank the Reviewer for this suggestion and have added cartoons to indicate whether different Split-GAL4 lines express in TPN1 and/or VNC-SP neurons in Fig 2 and Fig S5-S8.

3. If you invert the position of panels A and BC in figure 6 then you can remove the question mark from your figure and give more weight to your findings.

We thank the Reviewer for this suggestion and have adapted figure 6 (now Figure 5 in this resubmission accordingly).

Giorgio Gilestro, Imperial College London
