# Supplementary material for: Regulation of sleep by cholinergic neurons located outside the central brain in Drosophila
Source: PLoS Biol. 2023 Mar 2;21(3):e3002012. doi: 10.1371/journal.pbio.3002012 (PMC10013921; doi:10.1371/journal.pbio.3002012)
Supplement: Attachment — Submitted filename: Response to Reviewers.docx [file pbio.3002012.s026.docx]

**Reviewer #1:** The authors have addressed the few comments that I had, other than my point 1. I asked for a cartoon depicting the sleep system in flies, to help the non-expert reader along. Their answer is that "a cartoon that illustrates the multiple sleep-regulating regions in the fly brain would add confusion. ". This doesn't make any sense to me, why would adding a schematic cartoon add confusion. Anyhow, minor issue perhaps. I have no objections to the publication of this study at this stage.

However, I remain somewhat sceptical regarding the novelty of the findings and the appeal to the broad readership of PLoS Biology.

We thank the Reviewer for their review and have now added a cartoon depicting the sleep system in flies in Figure 1A.

**Reviewer #2:**

I find the authors' responses very clear, detailed and useful. I have these additional minor comments for the authors to consider. I think points 4/14 are the most important - discussion of the possibility that peripheral neurons/cells in the 23E10Gal4//23E10DBD lines (distinct from bowtie neurons) are sleep-promoting.

We thank the Reviewer for their review, comments, and suggestions.

1. Line 67 Remarkably,....

recommend you delete this word

We have deleted this word.

2. Line 71 "... we sought …. "

The historical backdrop is not the obligatory basis for the narrative. I suggest the intro should finish much more strongly - emphasizing the significance of the effort/results.

We thank the Reviewer and have rewritten this section to now read:

“In this study, we sought to identify the 23E10-GAL4 dFB neurons that are important for sleep regulation by conducting a targeted, intersectional Split-GAL4 (33) screen focused on 23E10-GAL4 dFB neurons. We report here that the 23E10-GAL4 driver expresses in many non-dFB neurons in the brain and in the Ventral Nerve Cord (VNC), the fly equivalent to the spinal cord. Furthermore, we have identified two non-dFB 23E10-GAL4 expressing, sleep-regulating cholinergic neurons located in the VNC. Our analyses reveal that thermogenetic and optogenetic activation of these neurons promote sleep while silencing increases wakefulness. In addition, we provide evidence that these VNC sleep-promoting neurons are not involved in sleep homeostasis while other 23E10-GAL4 expressing cells are. Thus, we conclude that 23E10-GAL4 contains at least two different types of sleep-regulating populations, the two VNC neurons we are describing in this study and another group, likely made of dFB neurons, that regulate sleep homeostasis.”

3. Line 146 "... and is widely considered as a "dFB-specific" tool."

This is an un-referenced observation - who says so and how widely-considered?

We thank the Reviewer for spotting the lack of references for this statement. It turns out that 23E10-GAL4 has been used as a dFB driver in more than 30 published studies. We have added several references supporting our statement in the revised manuscript.

4. Line 152. "Importantly, 23E10-GAL4 does not express in the legs, gut, or ovaries (Fig S1B-S1E) indicating that its sleep-promoting properties originate in the CNS."

Based on that, the authors state: 'We found that 23E10-GAL4 does not express outside the CNS (Fig. S1B-S1E).'

This conclusion is clearly premature; the survey of the periphery is largely incomplete. This is best done by staining sections of intact Drosophila. This is not a trivial point to my mind. The authors have argued effectively that the 23E10Gal4 line contains sleep-promoting cell types in addition to the dFSB ones. They showed that you must look outside the brain to find them and they found candidates in the ventral nerve cord. They were able to implicate 2 of those neurons by also finding them in different split gal4 lines which contained the same 23E10 DBD activity. I think the same logic that drove the authors to shine light on the bowtie neurons as candidate sleep regulators should prompt them to ask whether additional ones (or maybe even the "real" non-brain 23E10 sleep promotors) reside outside the CNS. If these exist, they may inextricably linked to the bowtie neurons in terms of enhancer activity, for the regulatory sequences tested. They should at least concede such neurons may exist. The finding of others (cited as #33) that leg cells have sleep-promoting activity serves as a clear precedent. (Also see point #14 below).

Also, I recommend you delete the word "Importantly,"

We agree with the Reviewer that in principle, it is possible that there may be sleep-promoting 23E10-GAL4 expressing cells outside the CNS in tissues we have not assessed in this study. We think however that this is extremely unlikely. See also our responses to points 14 below. We have modified this section to now read:

“Finally, since a recent study has implicated neurons located in the legs in sleep homeostasis (38), we also examined expression in the legs, gut, and ovaries in *23E10-GAL4>UAS-GFP* flies. Our analysis revealed no GFP staining in these structures (Fig S1B-S1E). While it is possible that 23E10-GAL4 expresses in other non-CNS cells that we have not assessed in this study, we feel that this is extremely unlikely. It is even more unlikely that these non-CNS neurons would regulate sleep. Thus, we conclude that the sleep-promoting properties of the 23E10-GAL4 driver probably originates in the CNS.”

5. Line 151 "….these results indicate that it is impossible to unequivocally assign a role in sleep promotion to 23E10-GAL4 dFB neurons (Fig 1I)."

I think it fair at this point to re-visit the original Donlea et al experiments in which dFSB represented the overlap of three different positive Gal4 lines. Such independent application of different driver lines was not done here. Instead the 23E10 DBD line has properties equal to the original 23E10 Gal4.

We feel that our statement here is accurate. Our comment only pertains to the use of 23E10-GAL4 as a driver to manipulate the dFB, which is what many laboratories are doing. We only want to emphasize the fact that if one chooses to use 23E10-GAL4 as a driver to manipulate the dFB “specifically” and obtains sleep phenotypes, it is simply impossible to conclude that these are the results of a dFB specific manipulation. This fact becomes even more evident with the identification of VNC-SP neurons.

We are aware that the *Donlea et al*. study used three different GAL4 lines. They all have expression in the dFB, but there is no data looking at whether the dFB cells contained in one of these drivers are the same as those contained in the others. In addition, these drivers are widely expressed throughout the brain and the VNC, and no assessment was made to identify potential neurons outside the dFB that are present in all three drivers. Considering all of this, it is possible that these 3 drivers share expression in non-dFB sleep-regulating neurons, in addition to having expression in the dFB (which may or may not be common between these 3 drivers). Finally, we are talking about 23E10-GAL4 dFB neurons and we have no idea whether these are also present in the original three drivers identified by the Donlea study.

In addition, we provide new data demonstrating that VNC-SP are also contained within the 23E12-AD; 23E10-DBD and VT020742-AD; 23E10-DBD Split-GAL4 lines (Fig S11 and Fig S14), and that they are sleep-promoting neurons in these two other lines. Thus, we have identified 3 different lines that express in VNC-SP neurons and are sleep-modulating.

6. Line 195: the 23E12-AD; 23E10-DBD Split-GAL4 "Surprisingly, our anatomical analyses revealed that it does not express in any dFB neurons (Fig 1O, Movie S3, Movie S4). Instead, this line expresses in two clusters of 4-5 neurons located in the anterior ventrolateral protocerebrum and in 4 VNC cells located in the metathoracic ganglion."

Then on line 211:

"Importantly, since all the neurons contained in the 23E12-AD; 23E10-DBD Split-GAL4 are part of the 23E10-GAL4 expression pattern, these data indicate that this driver contains non-dFB sleep-promoting cells."

 Question: are the two clusters of 4-5 neurons located in the anterior ventrolateral protocerebrum in the original 23E10-Gal4? I infer from the conclusion that they known to be sleep-promoting

Yes, the two clusters of 4-5 neurons are present in 23E10-GAL4 but they are difficult to see in *23E10-GAL>UAS-GFP* flies as the signal from the dFB neurons and processes is extremely strong and overpowers the cell bodies and processes from the cluster cells. We have surrounded them on the left in what is Figure 1P. In addition, to further show these cell bodies, we provide a stack centered on these cells in a 23E10-GAL4>UAS-GFP brain below. Note that there may be more than 4-5 neurons in this region in 23E10-GAL4. Finally, we feel that there is confusion about the role of these two clusters of cells in sleep regulation. Our new data (Fig S11 and Fig S14) demonstrate that VNC-SP neurons are present in 23E12-AD; 23E10-DBD, and that they are sleep-promoting. We have not investigated these two clusters of 4-5 neurons in sleep regulation, so we do not know if they are involved or not.

7. Line 206 23E12-AD; 23E10-DBD additionally labels two neurons in the VNC whose somas are located very close to the "bowtie" neurons. These neurons have characteristic processes in each leg ganglion.

How was it determined whose processes are whose, among the 4 VNC neurons?

We have added a side view of the VNC in Figure 1P. The processes of the “bowtie” neurons are easily distinguishable from those of the two TPN1-like cells. The processes of TPN1-like neurons stay close to the ventral side while the “bowtie” processes move dorsally in the VNC. There is a clear separation between the two types of processes making it easy to trace and follow them.

8. Line 211 Importantly

recommend you delete this word

We have deleted this word.

9. Line 236 Remarkably

recommend you delete this word

We have deleted this word.

10. Line 354 We will from now on refer to these neurons as VNC-SP (VNC Sleep-Promoting).

Is that necessary? Why not stay with Bowtie neurons? There could be additional VNC-SP.

There could also be additional Bowtie neurons. We think that VNC Sleep-Promoting (VNC-SP) better captures the role that these cells play in sleep regulation, and we would like to keep referring to them as VNC-SP.

11. Line 459 Importantly

recommend you delete this word

We have deleted this word.

12. Line 460 the weak RNAi line does not reduce sleep significantly -

I recommend you make this point more strongly - it is very good for your argument.

We thank the Reviewer for this suggestion, it now reads “The second ChAT RNAi line (25856) only modestly reduces total sleep in female flies, when expressed in VNC-SP neurons. However, these flies show a significant increase in waking activity, suggesting that the sleep reduction may be caused by hyperactivity. No other sleep parameters are changed when expressing this weakest RNAi line in VNC-SP neurons in males or females (Fig S13J-S13Q). These behavioral data agree with our quantification of RNAi efficiency (Fig S13C-S13E), with only the stronger RNAi line (60028) capable of significantly modulating sleep when expressed in VNC-SP neurons.”

13. Line 528 One of the goals of neuroscience is to ascribe behaviors to individual neurons.

Maybe refine this statement? Individual neurons are not absolute agents of control in all animals. Maybe 'ascribe to specific cell types'?

We have modified the sentence to now read “One of the goals of neuroscience is to ascribe behaviors to specific cell types.”

14. Line 539 Also unclear was whether the sleep promotion caused by activation of 23E10-GAL4 neurons is due to dFB neurons or to other cells within the 23E10-GAL4 pattern of expression.

Again, I think this is over-stating the case against the original conclusions: three independent and different Gal4 lines were positive in that original screen - they shared the dFSB as a common point of expression in the brain. The present work has extended focus to the bowtie neurons of the VNC. But by the author's logic, it is fair to say it remains unclear whether the sleep promotion (caused by activation of the split Gal4 lines they have used) is due to BT neurons, or due to other (peripheral?) cells. (related to point #4 above).

Similarly to our response to comments 4 and 5 above, we feel that our statement is accurate. We cannot comment on the original three GAL4 drivers used to involve the dFB in sleep regulation. We are specifically talking about the 23E10-GAL4 dFB neurons which may or may not be the same dFB neurons contained within the 3 original GAL4 drivers. We have identified three different Split-GAL4 lines (all based on 23E10-GAL4) that express in VNC-SP neurons and that modulate sleep. For the cleanest line (VT013602-AD; 23E10-DBD) that only expresses in the two VNC-SP cells, we included new data showing that this line does not express in the legs, gut, or ovaries (Fig S5D). Furthermore, we demonstrate that removing VNC-SP cells from the pattern of expression of these 3 Split-GAL4 lines effectively blocks the sleep induction (Fig S13R-S and Fig S14). Thus, we are extremely confident that VNC-SP are the sleep-promoting neurons in all our 3 lines.

15. Line 636	Note that a similar approach was used to validate ChAT RNAi constructs in other studies.

What other studies?

We thank the Reviewer for pointing out the lack of reference for this statement. We have now added the reference (30): Ni JD, Gurav AS, Liu W, Ogunmowo TH, Hackbart H, Elsheikh A, et al. Differential regulation of the Drosophila sleep homeostat by circadian and arousal inputs. Elife. 2019;8.

16. Fig 4 BC

There appear to be more than 1 BT neurons on each side. I do not see clear positive/negative signals for NT stains in the GABA panel.

We only see one VNC-SP neuron per side. We changed the GABA panel in Figure 4C to reflect that fact more clearly and to provide positive/negative GABA staining.

Reviewer #3: The authors have addressed pretty much all the important issues raised by the reviewers and I think produced a manuscript ready for publication.

We thank the Reviewer for their review.
